# A Treatise on Tetanus; Being the Essay for Which the Jacksonian Prize, for the Year 1834, Was Awarded by the Royal College of Surgeons, London

**Published:** 1837-04

**Authors:** 


Art. I.—*A Treatise on Tetanus; being the Essay for which the Jacksonian Prize, for the year* 1834, *was awarded by the Royal College of Surgeons, London.* By Thomas Blizard Curling, Assistant Surgeon to the London Hospital, and Lecturer on Morbid Anatomy.—*London,* 1836. 8vo. pp. 236.

This work is the result of careful compilation, patient enquiry, and judicious reflection. It neither achieves nor aims at much originality; yet it contains some valuable additions to our knowledge of tetanus. We are not disposed to admit that the systematic accounts of this disease hitherto published are so imperfect as Mr. Curling considers them to be; more especially since the publication of Dr. Symonds's excellent article in the Cyclopædia of Practical Medicine, which we are surprised not to find referred to by him; still, as the best and most recent treatises have appeared in general collections of medicine, there was ample room for an independent work like the present.

In the description of this disease by Mr. Curling, we meet with little that may not be found elsewhere; but the author deserves credit for directing attention to the spasmodic contraction of the muscles of the glottis, which would appear to be not unfrequently the immediate cause of death. Some interesting statistical information is collected partly from the observations of M'Grigor, O'Beirne, Howship, Blane, and Dickinson, and partly from a table of 128 cases of traumatic tetanus, arranged by Mr. C. in such a form as to show the age and sex of the patients, the nature of the injury, the treatment, and the termination of the disease. It appears that the disease is equally fatal at every period of life, from the age of ten to forty-five; but its frequency and fatality are much greater in the male than the female sex.

"Of the 128 cases in the table, sixteen were females, being to the males in the proportion of one to eight. Of these sixteen cases, four only were fatal; whereas, out of the 112 cases of males, sixty-six, or more than one-half, died. The comparative rarity of tetanus in women, is partly accounted for by their being less exposed, both to the predisposing and to the exciting causes of the disease; and, since it is observed to occur more frequently in persons of a robust constitution and of great muscular strength, females are supposed to be less susceptible of it." (P. 29.)

The statements respecting the period of accession, and the duration of the disease, are identical or correspond with those of former writers upon the subject. The sections which treat of the causes, prognosis, and diagnosis, present nothing of sufficient interest to detain us, though they are marked by the same carefulness of statement and of inference as are the other parts of the book.

The most important chapter is that devoted to the Pathology of Tetanus. The author first describes the various morbid appearances observed in the cerebro-spinal axis, the sympathetic system, the nerves immediately connected with the seat of injury, the muscular system, the viscera, and other parts of the body; and in doing this he exhibits no lack of research. But the facts thus collected are insufficient in themselves to afford a clear theory of the disease, the morbid changes being of the most variable description; neither peculiar to tetanus, nor observable in every specimen of this affection. Taken, however, in connexion with the phenomena during life, and with our knowledge of the physiology of the nervous system, they assist (perhaps as much by their negative as their positive characters,) in the formation of an approximative estimate of the essential nature of the disease. The following are the general conclusions at which Mr. Curling has arrived upon this subject: (1.) That the disease is functional; a peculiar irritation, independent of inflammation or any structural alteration. (2.) That the seat of this irritation is the *tractus motorius* on either side. (3.) That the result of it is "the supply of a stimulus to abnormal action" to the voluntary muscles. (4.) That it is excited either by an impression propagated to the medulla from distant nerves, (most probably sentient,) or by inflammation in the brain, spinal cord, or their membranes; the inflammation having either commenced in these organs, or extended continuously from the nerves of a wounded part. (5.) That "the primary impression is confined for an indefinite time to the nerves of the part injured." (6.) That the irritation in the medulla, once excited, continues independently of the exciting cause. (7.) That "tetanic irritation often gives rise to a determination of blood to the cord and its meninges, and to the nerves proceeding from the site of the wound and to the affected muscles; the result of which, in the medulla, is an increase in the natural secretion of the arachnoid. The minute vascular injection of the cord and of the nerves, together with the serous effusion at the base of the brain and between the spinal membranes, being therefore nothing more than occasional effects of the disease, are by no means constantly present after death."

We have omitted two or three of Mr. Curling's general inferences, because we are pressed for room, and they are of less interest than those which we have mentioned.

After a review of the various plans of treatment, and of the individual remedies which have been resorted to for the relief of tetanus, the author very justly observes, that, as there are different forms of the disease, so they each require a different kind of management. He distributes his general directions under the heads of—1. Pure acute Tetanus; 2. Acute inflammatory Tetanus; 3. Chronic Tetanus. The second of these must, it is obvious, be treated upon strict antiphlogistic principles in the commencement, though it may subsequently require the application of measures similar to those adopted in the pure tetanus. The means employed in the chronic form, whether this be inflammatory or purely tetanic, differ more in degree than in kind from those demanded by the acute varieties. We shall quote what is said upon the treatment of the first form:

"*Pure Acute Tetanus.* If the disease be traumatic, and the patient is seen at the very outset, topical means may be applicable. The medical treatment of this form mainly consists in maintaining a free action of the bowels, in allaying the spasms with the tobacco, cold affusion, or any other sedative equally effective; and in the due exhibition of tonics and stimulants. Here success, especially in cases attended with impending suffocation, chiefly depends upon the energy, perseverance, and judgment with which the necessary means are employed. No time must be lost in the trial of inactive remedies, or of such as experience has demonstrated generally to fail. From the period that the patient is first seen, he should never be left until the spasms are in a great degree relieved. If opium be tried, unless its influence be speedily produced and the muscles relaxed, it must be at once abandoned. By the use of cold affusion, or of tobacco, the spasms can generally be controlled. These remedies, therefore, must be vigorously employed, and persevered in, until the required effects are obtained. Brandy and ammonia should be at hand; and, as soon as reaction is established, and the paroxysms return, these sedative means must be again resorted to. Tobacco being more manageable, and, if employed with sufficient care, unattended with danger, is preferable to cold affusion, which might only be used when the other remedy is unattainable."

The last chapter is occupied with Tetanus Nascentium; and the volume concludes with (as far as it goes,) a well-selected Bibliography. Upon the whole, we do not hesitate to say that Mr. Curling's work is likely to be very acceptable to the profession, and that, in our judgment, it reflects credit upon the Jacksonian institution, which called it forth.

